# Cost and Cost-Effectiveness Assessments of Newborn Screening for Critical Congenital Heart Disease Using Pulse Oximetry: A Review

**DOI:** 10.3390/ijns3040034

**Published:** 2017-12-14

**Authors:** Scott D. Grosse, Cora Peterson, Rahi Abouk, Jill Glidewell, Matthew E. Oster

**Affiliations:** 1 Centers for Disease Control and Prevention, National Center on Birth Defects and Developmental Disabilities, 4770 Buford Highway NE, Mail Stop E-87, Atlanta, GA 30341, USA; 2 Centers for Disease Control and Prevention, National Center for Injury Prevention and Control, Atlanta, GA 30341, USA; 3 Cotsakos College of Business, William Paterson University, Wayne, NJ 07470, USA; 4 Children’s Healthcare of Atlanta, Emory University School of Medicine, Atlanta, GA 30341, USA

**Keywords:** neonatal screening, critical congenital heart disease, economic evaluation, cost-effectiveness, health policy

## Abstract

Screening newborns for critical congenital heart disease (CCHD) using pulse oximetry is recommended to allow for the prompt diagnosis and prevention of life-threatening crises. The present review summarizes and critiques six previously published estimates of the costs or cost-effectiveness of CCHD screening from the United Kingdom, United States, and China. Several elements that affect CCHD screening costs were assessed in varying numbers of studies, including screening staff time, instrumentation, and consumables, as well as costs of diagnosis and treatment. A previous US study that used conservative assumptions suggested that CCHD screening is likely to be considered cost-effective from the healthcare sector perspective. Newly available estimates of avoided infant CCHD deaths in several US states that implemented mandatory CCHD screening policies during 2011–2013 suggest a substantially larger reduction in deaths than was projected in the previous US cost-effectiveness analysis. Taking into account these new estimates, we estimate that cost per life-year gained could be as low as USD 12,000. However, that estimate does not take into account future costs of health care and education for surviving children with CCHD nor the costs incurred by health departments to support and monitor CCHD screening policies and programs.

## 1. Introduction

Newborn screening (NBS) can save both lives and healthcare costs, although testing for any given condition may accomplish only one or the other [1]. However, the up-front costs and logistical challenges of instituting screening of all or almost all newborn infants, few of whom will be found to be affected, can deter policy makers from instituting new screening programs or adding conditions to an existing program. The economic balance between the costs and benefits of screening has long been recognized as a desirable attribute of population screening programs [2]. Although some jurisdictions require a prospective economic assessment to inform decisions on the expansion of NBS [3], screening policy decisions have often not required a demonstration of cost-effectiveness in practice [4,5].

Although most NBS is done through centralized laboratory analyses of dried bloodspot specimens collected from newborns, point-of-care NBS for certain conditions is typically performed before discharge from the birthing facility [6]. In particular, screening for various types of critical congenital heart disease (CCHD) (Table 1) can be done using pulse oximetry, a simple, non-invasive test for hypoxemia in which sensors measure blood oxygen saturation through light passing through the skin [7]. Neonatal hypoxemia can have either cardiac or non-cardiac causes. When the US Department of Health and Human Services added CCHD to its Recommended Uniform Screening Panel in 2011, it requested the Centers for Disease Control and Prevention (CDC) to conduct a cost-effectiveness analysis (CEA) of newborn screening for CCHD [8,9]. That information was intended to inform policy decisions by state governments. In 2013, CDC researchers (Peterson et al.) published the first full CEA of CCHD screening in the US setting [10]. Although previous studies had estimated the cost of screening and the cost per case of CCHD detected [11–17], “Calculating the cost to detect a case tells one nothing about the value of detecting and treating the disease in question and, hence, is not informative of the balance of costs and outcomes” [18]. A full CEA includes estimates of the numbers of deaths averted and avoided healthcare costs associated with prompt diagnosis. Peterson et al. reported a point estimate of roughly USD 40,000 in net cost per life-year saved, a figure consistent with commonly used cost-effectiveness thresholds [19]. More recently, a CEA assessed the potential cost-effectiveness of CCHD screening in different localities in China, reporting that screening could be cost-effective in some settings [20].

The present review summarizes and critiques previously published estimates of the costs or cost-effectiveness of CCHD screening—two UK studies in four publications [12–14,17], three US studies in four publications [10,21–23], and one study from China [20]. Updated estimates of costs, outcomes, and summary measures of the economic value of CCHD screening in the United States are also presented. The new estimates address future disease-related treatment costs, shortened life expectancy among CCHD survivors, and a higher estimated number of avoided infant deaths due to screening.

## 2. Review of Previous Estimates

CEA studies of screening programs or policies require as inputs, estimates of the costs of screening, diagnosis, and intervention, numbers of cases detected in a timely manner as a result of screening, differences in health outcomes with and without screening (i.e., effectiveness), and differences in healthcare and other costs for affected individuals between scenarios with and without screening [18,24]. Each of those parameters is context-specific; therefore, it is difficult to use a given study to generalize as to whether screening may be cost-effective in different contexts. For example, a given screening test may have very different costs and outcomes in different settings depending on the characteristics of the population and how screening is implemented in that setting. In addition, the assessment of cost-effectiveness depends on how much decision makers are willing to pay for an intervention or program in relation to the expected health gains. This means that different stakeholders and jurisdictions that consider the same evidence on costs and outcomes may reach different conclusions about economic value. Therefore, instead of asking whether screening is cost-effective, it may be more constructive to ask when and by whom screening might be considered cost-effective [24,25].

The analytic perspective of an economic evaluation must be specified. From the societal or healthcare sector perspectives, cost refers to the opportunity cost of resources used up in providing a service rather than the direct financial outlay or expenditure of money. Many CEAs of clinical interventions take the healthcare sector (or system) perspective, in which only formal healthcare sector costs are considered [26,27]. A societal perspective analysis should also include informal healthcare costs, such as the time and expenses incurred by patients and families, non-health sector costs such as educational expenditures, and productivity losses resulting from death or premature mortality [28]. Spillover effects of illness or disability on the health of family members and on the time use and economic productivity of family caregivers are also germane for societal-perspective cost-effectiveness analyses [29]. The societal perspective is particularly appropriate for the analysis of public health policies and programs, and such analyses should include the costs of public health activities in support of clinical interventions.

### 2.1. Cost of Critical Congenital Heart Disease (CCHD) Screening and Follow-Up

The one element common to all previous economic evaluations of CCHD screening is inclusion of the directly estimated cost of screening, which is readily measurable for CCHD screening. Most studies employed a micro-costing approach with separate estimates for staff time, instrumentation, and consumables (Table 2). An exception is a cost-effectiveness study from China that did not report detailed screening costs [20]. The micro-costing estimates from other studies are summarized below separately for labor (Section 2.1.1) and instruments and consumables (Section 2.1.2). In addition to the direct costs of pulse oximetry screening, the incremental cost of CCHD screening includes the costs incurred by the healthcare system for the follow-up and management of patients who screen positive. That includes clinical examinations and diagnostic tests attributable to screening. It also includes the differences in treatment costs that result from early diagnosis. Estimates for those cost components are summarized in Sections 2.1.3 and 2.1.4.

In principle, the full spectrum of a screening program or policy also includes the costs of a public health program in coordinating the implementation of screening, reporting of screening results, short and long-term follow-up of screen-positive patients, and the surveillance or tracking of short and long-term outcomes. However, no estimates of public health costs associated with CCHD screening have been published to date.

#### 2.1.1. Labor Cost

The cost of staff time per screen is the product of the average number of minutes per screen and the average wage or labor cost per minute (see Table 2). Empirical screening time estimates can be obtained by either asking nursing staff to estimate their own time or by conducting direct observation, i.e., time-and-motion studies. Three time-and-motion studies conducted in birthing hospitals in three US states using the same data collection methods all calculated that nursing staff took on average 9–10 min per newborn to complete a CCHD screen, including preparation and paperwork [21,23,30]. A similar UK study estimated an average time of 7 min per newborn, but preparation time was minimized because parental consent had been obtained before birth [12]. In contrast, studies based on clinical opinion have assumed that screening takes 2–5 min per newborn [11,13,14,31]. Self-reports by nursing staff in two US hospital-based studies reported average screening times of 3.5–5.5 min per newborn [22,32], although that does not include time spent explaining results to families or documenting screening results [22].

The per-minute cost of staff time for CCHD screening is a function of both the pay scale in a given population and the pay grade of the staff who perform the screening, which can vary by clinical setting and time period. Most programs use registered nurses or midwife staff in a newborn unit. Cost estimates from older studies that used research assistants or physicians to conduct screening do not reflect current hospital procedures. Another source of variability in estimates, at least in US studies, is differences in how labor costs were calculated. Some US studies have considered only hourly wages [22], whereas others included total hourly compensation, including taxes and fringe benefits [21,23]. The most recent UK study cited an official database for unit staff costs [12]. An earlier UK study included an adjustment for administrative overhead as well as benefits [14]. Hourly labor costs differ by occupation and geographic labor market; labor cost per minute was roughly three times higher in a US study conducted in New Jersey [21] than in one conducted in Utah [23] using similar methods (Table 2).

#### 2.1.2. Instruments and Consumables

The equipment cost of CCHD screening consists of the amortized costs of the pulse oximeter machine and reusable probes or sensors and the consumable cost of disposable probes or probe tips and straps. The variety of probe types used for screening creates great heterogeneity in cost estimates. Variable cost per newborn is very low when a reusable probe is used for screening compared to the use of a disposable probe, although there are maintenance costs with reusable probes. A US study conducted by Peterson et al. in a statewide sample of hospitals in New Jersey estimated that the equipment cost per infant screened was $0.49 using fully disposable probes, $1.74 using reusable probes with disposable tips, and $13.62 using disposable probes (2014 USD) [21]. A subsequent US study conducted in two facilities in Utah suggested that the total equipment cost per newborn was $21.92 using disposable probes and $0.25 using reusable probes (2014 USD) [23] (Table 2). British economic evaluations have assumed the use of the less expensive reusable probes [12,14,17].

#### 2.1.3. Diagnostic Work-Up

If a newborn does not pass CCHD screening, the newborn is referred for a diagnostic work-up to determine the cause of hypoxemia, whether cardiac or non-cardiac. The diagnostic process may include a clinical evaluation, laboratory and/or imaging tests (including echocardiography), and possibly transportation to another hospital for further evaluation and management. The diagnostic work-up for non-cardiac causes is variable, and no published studies of CCHD screening to date have included estimates of clinical examination to identify potential non-cardiac causes of hypoxemia. Previous UK studies of CCHD screening costs assumed that newborns who do not pass CCHD screening would receive a 30 min diagnostic echocardiographic assessment by a pediatric cardiologist at a cost of roughly £115 pounds per child (2009 GBP) [12,17]. No cost was assigned in that study for transportation.

Peterson et al. estimated costs of echocardiography based on administrative data from a private insurance claims database, with the analysis restricted to inpatient services during infancy. The cost estimate was a truncated arithmetic mean after excluding the top and bottom 1% of observations [10]. Based on data from Florida, indicating that roughly 43% of infants with CCHD were transferred to another hospital during the birth hospitalization [33], it was assumed that 43% of infants who screened positive would require ambulance transport for transfer to a hospital with echocardiography. The cost of ambulance transport was derived using the same methods as the cost of echocardiography [10]. The additional cost of diagnostic echocardiography and transport was estimated to add $0.25 per newborn screened (2011 USD).

#### 2.1.4. Treatment

The potential reduction in treatment costs associated with timely CCHD diagnosis is challenging to estimate, and only one study to date attempted to do so. In an analysis using linked Florida birth defects surveillance and hospital discharge data from 1998 to 2007, Peterson et al. estimated attributable hospitalization costs in the first year of life for infants with CCHD (*n* = 3603) [33]. Unadjusted costs were higher for infants with CCHD detected prior to discharge from the birth hospitalization than those detected later because severely affected infants are more likely to be detected sooner and to incur more costly treatment. In a statistical analysis that adjusted for CCHD type, maternal race/ethnicity, and other variables, late CCHD detection was found to be associated with 52% more admissions, 18% more hospitalized days, and 35% higher inpatient costs during infancy relative to the early detection of CCHD [33].

The CEA by Peterson et al. projected the avoided cost of hospitalization for infants with screening-detected CCHD in two steps. First, the difference in number of hospitalized days between infants with timely and late detected CCHD was derived from a previous analysis of Florida data [10,33]. Second, average hospital costs per day among infants with selected CCHD conditions were estimated, assuming that the cost per day was the same for early-detected and late-detected cases [10]. The underlying CEA model assumed that with screening, 77.5% of infants with CCHD would be detected prior to discharge, with an average hospital cost during infancy of $156,501 compared to $181,775 for those not detected, a difference of $24,677 per timely-detected CCHD case (2011 USD). Comparing screening and no-screening scenarios, the hospitalization cost was calculated to be $19,111 per infant with CCHD under screening, taking into account false negatives. That amount was equal to $7.48 per newborn infant (2011 USD) [10].

### 2.2. Screening Cost per Case Detected

Besides the direct cost of performing screening, it is straightforward to calculate the number of additional cases of CCHD detected in a timely manner due to screening. However, this number must be assessed in a given population, because the numbers of new CCHD diagnoses that can be made through the follow-up of screening using pulse oximetry is dependent on the use and accuracy of prenatal screening and postnatal clinical detection in that population. In particular, an increased frequency of prenatal diagnosis of CCHD results in fewer additional diagnoses of CCHD following CCHD screening using pulse oximetry [34]. The frequency of delayed CCHD diagnoses in the United States was shown to have decreased over time [35], although no decrease in post-discharge CCHD diagnoses was found in a study from the United Kingdom [36].

Multiple studies have estimated the screening cost per CCHD case detected, with widely varying estimates. For example, UK estimates range from roughly £5000 (2001 GBP) [13] to £24,000 (2009 GBP) per CCHD case detected [12] and US estimates range from approximately $21,000 (2011 USD) per case with timely diagnosis [10] to $46,000 (2012 USD) per case detected [22]. Different assumptions about two cost elements—the cost per newborn screened and the averted costs of care resulting from timely diagnosis—account for much of this variation. In particular, some estimates are of gross screening costs and others are net screening costs, after subtracting the avoided cost of treatment. For example, Kochilas et al. reported a relatively high cost per case detected ($46,000), despite assuming a low screening cost [22]. In contrast, Peterson et al. assumed a higher cost of CCHD screening and reported that screening would cost approximately $21,000 per case with timely diagnosis [10]. The Peterson et al. estimate reflected the *net* cost of screening, after subtracting the estimated savings in hospitalization costs during infancy resulting from timely diagnosis, which offset more than half of the screening cost. The gross cost per case detected in that study was roughly $46,000.

As noted in Section 2.1.3, the choice of reusable or disposable pulse oximeter probes can have an even larger impact on estimated costs of CCHD screening. Peterson et al. assumed average equipment and labor costs of $6.86 and $6.62 per infant, respectively (2011 USD) [10]. The equipment cost was derived from a sample of seven New Jersey hospitals, only one of which used fully reusable probes. If it had been assumed that all hospitals used fully reusable probes, the average equipment cost would have been much lower [21], and the average cost per newborn screened would have been roughly half as great.

### 2.3. Health Gains

It is challenging to estimate health gains from CCHD screening, which encompass avoided early deaths and improved health outcomes among survivors. Cost-effectiveness guidelines call for both types of health outcomes to be expressed in summary measures of health such as the quality-adjusted life-year (QALY) that take into account changes in life expectancy and morbidity [26–28]. However, evidence of improved health among survivors following timely diagnosis is lacking. In addition, it is challenging to find conceptually and empirically appropriate estimates of utility weights for pediatric conditions that can be used to project QALY gains [37–39]. Consequently, it is common for CEAs of interventions that primarily affect survival to calculate the cost per life-year saved, since it makes little practical difference in ranking the cost-effectiveness ratios for such interventions [40].

Two CEA studies published estimates of numbers of deaths avoided through CCHD screening. First, Peterson et al. conservatively assumed that CCHD screening, if applied to four million US births each year, would avoid 20 infant deaths [10]. This estimate was based on a national estimate of 28 annual deaths associated with delayed diagnosis of CCHD in the absence of universal screening. The estimate of 28 deaths was derived by multiplying four million births by an avoidable mortality rate among infants with late-detected CCHD of 1.8% and a frequency of 38.8 infants with late-detected CCHD per 100,000 births, both calculated from a study of linked births and deaths in Florida prior to the introduction of screening [33]; the overall preventable death rate among infants with CCHD was 0.4%. To calculate the number of life-years saved as a denominator for a cost-effectiveness ratio, Peterson et al. took the estimated number of infant deaths averted by screening and multiplied this by 30.7 years, which was the life expectancy at birth of the US population discounted to the present with a 3.0% discount rate per year [10]. In other words, the investigators assumed that surviving infants would have a normal life expectancy.

Tobe et al. estimated infant deaths from CCHD for three scenarios in three Chinese cities [20]. The base scenario assumed that infant mortality would be the same as in the 1950s (standard practice in CEAs is to use recent estimates as the baseline to compare with an intervention). The authors also assumed that “that infant mortality would reduce to approximately 25% if infants received timely diagnosis and treatment”, but did not state the assumed mortality rates with and without screening. The ratio of assumed averted deaths to CCHD cases was 0.225 in Beijing, 0.112 in Shandong, and 0.046 in Gansu. They assumed that access to timely diagnosis and treatment varied across regions (e.g., apparently one-half as high in Shandong as in Beijing), but did not make those assumptions explicit. Tobe et al. also reported outcomes in terms of disability-adjusted life-year (DALY) estimates, a measure analogous to the QALY, but did not explain how the DALY estimates were generated [20]. It should be noted that the “disability” weights used to calculate DALYs refer to “any short-term or long-term loss of health” and hence assess morbidity rather than disability [41].

### 2.4. Cost-Effectiveness Ratios

It is standard practice for CEAs to report incremental cost-effectiveness ratios (ICERs) for interventions that are found to be both more costly (net positive cost) and to improve health outcomes relative to next most cost-effective strategy analyzed. To calculate the numerator of the ICER, analysts are supposed to subtract any cost offset associated with improved health from the cost of the intervention. The denominator is the difference in the number of health outcomes attained, most commonly in the form of discounted life-years or health-adjusted life-years gained relative to the alternative. In some countries, policy makers set threshold values or ranges for ICERs below which interventions are presumed to be considered cost-effective. For example, thresholds for approval of the coverage of new medications have been estimated at roughly £30,000 (GBP) per QALY in the United Kingdom, CAN$50,000 in Canada, and AUS$42,000 in Australia [42].

Peterson et al. projected that the point estimate of the net cost of screening was just over $40,000 per life-year saved in 2011 US dollars [10]. It is increasingly recommended that CEAs include probabilistic sensitivity analyses in which estimates for multiple parameters are allowed to vary simultaneously within ranges using probability distributions and simulation modeling techniques [43]. Peterson et al. also reported that a probabilistic analysis projected a one in three chance that CCHD screening would be cost-saving (i.e., negative net costs), and a roughly four in four probability that CCHD screening would cost less than $100,000 per life-year gained. It should be noted that there is no official threshold or standard for cost-effectiveness in the United States, although a private organization, the Institute for Clinical and Economic Review, has used a range of $100,000 to $150,000 per QALY to establish a “value price” for new therapies [44]. It should also be recognized, though, that because utility weights are usually less than 1.0 and decrease with advancing age, the number of QALYs gained is necessarily less than the number of life-years saved. Consequently, the cost per QALY gained is larger than the cost per life-year gained, often by at least 15–20%, which can affect the interpretation of ICERs relative to benchmark values [24].

CEAs are recommended to report sensitivity analyses that show the dependence of results on uncertainty in estimates or assumptions of model parameters [26–28]. Peterson et al. reported that the three variables with the greatest influence on the cost-effectiveness of CCHD screening were the reduction in hospitalization costs during infancy from early detection, the baseline proportion of late-detected CCHD cases, and the hospital cost of CCHD screening [10]. Variables that had the least influence on the results were the cost of echocardiography, cost and probability of transport for echocardiography and subsequent treatment, the mortality rate among infants with CCHD detected by screening, and the rate of false positives.

It should be noted that the assumption of 18% higher hospitalization costs with the late detection of CCHD assumed a fixed cost per hospital day. However, patients in critical care incur much higher costs per hospital day, and infants with late-detected CCHD are more likely to be in critical care units. The observed difference in hospitalization costs in infancy associated with late detection was twice as large as the difference in hospital days, 35% vs. 18% [33]. Therefore, the CEA was conservative; if the study had assumed 35% lower hospitalization costs in infancy with early detection, the cost-effectiveness ratio could have been close to zero.

Many CEAs reported in low- and middle-income countries use a threshold of three times per capita gross domestic product (GDP) per DALY, which was endorsed in the early 2000s by a World Health Organization report. Using that approach in China, with GDP per capita calculated by province, Tobe et al. concluded, “the intervention was highly cost-effective in Beijing (Int$7833/DALY); cost-effective in Shandong (Int$27,780/DALY); and not cost-effective in Gansu (Int$167,407)” [20]. However, those are average cost-effectiveness ratios rather than ICERs. The authors’ Table 1 indicates ICERs for CCHD screening of Int$15,020, Int$51,636, and Int$307,952, respectively. If the same arbitrary cost-effectiveness threshold had been applied to the ICERs, CCHD screening would not have been considered cost-effective in either Shandong or Gansu. In addition, it should be noted that the three times GDP per capita cost-effectiveness threshold value has been challenged as lacking validity [45].

## 3. Revised Cost-Effectiveness Estimates

### 3.1. New Estimates of Averted Infant Deaths

Newly published estimates of the impact of CCHD screening policies in the United States suggest that the actual reduction in CCHD deaths associated with universal screening may be much higher than previous economic evaluations anticipated. A statistical analysis of the association of US state CCHD screening policies that had been adopted by 1 June 2013 with the numbers of early infant (24 h to <6 months of age) deaths coded for CCHD or other/unspecified congenital heart defects (CHDs) through the end of 2013 found that the implementation of CCHD screening mandates was associated with a one-third lower frequency of recognized infant deaths with CCHD as the listed cause (see Supplement) [46]. The reduction in CCHD deaths was not entirely attributable to screening using pulse oximetry, since screening mandates could also have led to a greater clinical awareness and the earlier detection of CCHD using other methods. This reduction in CCHD-associated deaths was roughly six times greater than was assumed in the US CEA of CCHD screening by Peterson et al. based on the frequency of delayed CCHD diagnoses that could be avoided through screening [10]. Adjusting the Peterson et al. model to include 110 deaths averted annually (as opposed to the 20 deaths averted annually assumed in the Peterson et al. original study), the estimated cost per life-year gained becomes approximately $10,000 (2011 USD) (compared to the original reported estimate of approximately $40,000 per life-year gained).

### 3.2. Shortened Life Expectancy among Survivors

One factor unaddressed by Peterson et al. and other economic evaluations of CCHD screening is shortened life expectancy among individuals with CCHD. In other words, previous economic evaluations have implicitly or explicitly assumed that children with CCHD who survive infancy have the same life expectancy as unaffected children. However, all-cause mortality is higher among children with CHDs than for the general population, even after infancy [47]. For example, it has been widely reported that children and adolescents with CHDs have higher rates of cancer, even after excluding individuals with chromosomal disorders [48]. Survival with a CHD varies widely by condition or type of defect, country, ethnic group, and time period. Dramatic reductions over time in infant and early childhood mortality among cohorts of infants born with a CHD have been widely reported [49–52].

Few studies have reported survival probabilities for patients with CHDs relative to life-table survival probabilities for the general population. A population-based surveillance study conducted in metropolitan Atlanta, Georgia (USA), involving birth cohorts from 1979 to 2005, found that the average infant survival probability for infants with CCHD who had no non-cardiac defects or chromosomal syndromes was 75.2%, but increased from 67.2% during the first 15 years to 82.5% during the more recent 12 years [52]. One implication of the reductions in infant deaths associated with CCHD is a smaller number of deaths that could be potentially averted through screening. The average survival probability from 12 months to 25 years of age was 89.3%, which compares with a 99.3% survival probability for the general US population in the 2009 national life table [53]. A Danish study modeled post-surgery survival to age 25 among children born during 1990–2002 as 85% for all CHDs and from 64% to 87% for specific CCHD types, but the 10-year survival probability for the subsequent birth cohort was substantially greater, 93% vs. 87% overall [54].

It would be reasonable to project that life expectancy among individuals with CCHD who survive infancy could be 10–20% lower than in the general population. That would reduce discounted life-years by three to six years relative to population norms. Adjusting the US CEA model of CCHD screening reported by Peterson et al. to include 110 deaths averted annually and 20% fewer years lived yields an estimated cost per life-year saved of approximately $12,000 (2011 USD).

### 3.3. CHD-Related Future Medical Costs

It is generally recommended that CEAs include the present value of expected future medical costs for survivors whose deaths are avoided or postponed as a result of a healthcare intervention, although only one-half of CEAs published during 2008–2013 included estimates of future related medical costs [55]. Since infants with complex medical conditions incur substantial medical costs, including future medical costs can make life-saving interventions for such children appear less cost-effective. It is common for CEAs of newborn screening tests to not include future medical costs [40].

No estimates of lifetime medical costs for children with CHDs in general or for the subset with CCHD defects are currently available, with or without adjustment for co-occurring conditions. A US study that used linked birth defects surveillance and administrative data from California dating from the late 1980s estimated lifetime costs for four infants born with one of four specific CCHD conditions: truncus arteriosus, transposition of the great arteries/double-outlet right ventricle, tetralogy of Fallot, and single ventricle [56]. At the time of the study, infant survival was assumed to be low, with <50% of infants with truncus arteriosus or single ventricle reaching one year of age. A CDC publication recalculated the cost estimates with a 3% discount rate; lifetime medical costs per infant varied by condition from $86,000 to $260,000 (1992 USD) [57].

Given dramatic changes in survival and medical technologies, cost estimates from three decades in the past are not currently informative [58]. An analysis of 2005 US private insurance claims data reported that children with severe CHDs (roughly equivalent to CCHD) had mean expenditures during the first three years of life of roughly $210,000 in excess of children without CHDs [58]. The most recent estimates of hospitalization costs associated with birth defects estimated an aggregate burden almost twice as high in 2013 as in 2004 [59]. In 2013, the mean cost for a single inpatient stay with a CCHD diagnosis was $79,011, and aggregate hospital costs for CCHD patients of all ages generated a figure of $2.3 billion [59].

Despite the absence of recent estimates of lifetime costs for infants born with CCHD, it is possible to conduct sensitivity analyses. One potential proxy is the lifetime medical cost for infants born with spina bifida, which was recently estimated as $513,500 (2014 USD) [60]. In the analysis of California data from the late 1980s, medical costs for children born with spina bifida were similar to those born with single ventricle or tetralogy of Fallot and lower than those with truncus arteriosus [56,57]. However, annual medical costs for spina bifida remain elevated across the lifespan [60], whereas costs associated with care for severe CHDs drop off sharply after infancy. Roughly two-thirds of all hospital costs associated with CHD diagnoses are associated with admissions in the first 12 months of life [61]. Therefore, the lifetime medical cost for spina bifida might be an upper-bound estimate of the lifetime medical cost associated with CCHD.

Adjusting the US CEA model of CCHD screening reported by Peterson et al. to include 110 deaths averted annually, 20% fewer years lived, and $450,000 in post-infancy CCHD-attributable discounted future lifetime medical costs (as opposed to $0 included in the original study), yields an estimated cost per life-year saved of approximately $31,000 (2011 USD). That sensitivity analysis yields a conservative estimate of net benefit (i.e., the cost-effectiveness ratio is likely overestimated) since the actual incremental medical cost associated with CCHD is likely less than the proxy estimate that was used. In any case, the estimated cost is still lower than the originally reported estimate of approximately $40,000 per life-year gained.

## 4. Discussion

To date, all economic analyses of CCHD screening have followed the healthcare sector perspective, as is conventional for clinical interventions. All have included employee compensation costs to employers to estimate the cost of staff time. However, hospitals that have implemented CCHD screening have reported to be able to do so using existing nursing staff and do not incur additional staff costs [21]. From the hospital perspective, the cost of staff time might not need to be included [10]. From a societal perspective, the inclusion of staff time makes sense if nursing time used for CCHD screening could have otherwise been used for other tasks. If nursing time could not be reallocated, the time spent doing the screening would not represent an incremental cost. If that is the case, existing cost estimates could overestimate the incremental cost of CCHD screening.

Economic analyses of CCHD screening as a public policy should also include the costs of public health activities in support of CCHD screening and follow-up, but little is known about those costs. Jurisdictions implementing CCHD screening policies and programs often take varying approaches to the organization and surveillance of CCHD screening [62]. For that reason, it may not be possible to generalize the costs of the public health role in supporting and monitoring CCHD screening.

Notable variations exist in methods and results among the two previously published CEAs of CCHD screening [10,20] and several costing studies included in this review. The level of evidence for some cost elements, notably the cost of CCHD screening based on combined screening time, labor cost, instrumentation, and consumables cost, is reasonably established in some clinical settings. These cost elements still merit reconsideration or reinvestigation for future economic evaluations, for each is dependent on existing conditions in a given clinical setting. Estimates of effectiveness are also variable. Survival among infants with CCHD can vary greatly depending on location and treatment availability, which limits the generalizability of results across settings within countries, as well as across countries.

Other formal healthcare costs associated with CCHD screening are less well established. Some matter more than others, as indicated by their potential magnitude per infant screened. For example, the cost of the diagnostic work-up of screen-positive newborns is not well established, but given the very small numbers of infants who screen positive, this cost element contributes very little to the total cost of screening; perhaps 2% of the total cost [10]. It is likely that the assumption in the Peterson et al. study that 43% of newborns who do not pass CCHD screening require transport to another hospital is a substantial overestimate, but since such transportation contributed only marginally to the estimated total cost of screening in that study, modifying that assumption would have little effect on the results. In contrast, the cost of avoided hospitalizations associated with late-detected CCHD is highly influential [10]. However, that cost estimate in Peterson et al. was based on just one study in one US state conducted prior to the implementation of universal CCHD screening [33]. Furthermore, the Peterson et al. study assumed a high rate of sensitivity of CCHD screening.

Some healthcare costs have been neglected in economic evaluations of CCHD screening. One is the cost and benefits of diagnosing and treating non-CCHD cases among newborns referred following screening. The implicit assumption has been that the costs of diagnostic work-up relative to the size of the newborn population are low, similar to that of CCHD diagnosis. However, the potential health benefits of detecting and treating non-cardiac conditions, such as neonatal sepsis, deserve further attention. Non-health costs have been neglected in published cost-effectiveness analyses of CCHD screening to date. For example, no published information exists on the time costs incurred by family members caring for individuals with CCHD, nor the loss of economic productivity. Although there is evidence of the increased use of special education services among surviving children with CCHD [63,64], the associated costs have not been calculated. Furthermore, it is unknown whether early detection might influence long-term academic outcomes among those children.

Three parameters can substantially affect the overall CEA results and remain underinvestigated: estimates of averted infant CCHD deaths, shortened life expectancy among children with CCHD who survive infancy, and future CCHD-associated medical costs. Previous CEAs have relied on indirect estimates of averted deaths based on estimates of numbers of infant deaths with CCHD, the frequency of delayed diagnosis, and the assumed relative reduction with timely diagnosis; they have excluded the other two parameters. For example, Peterson et al. calculated an expected number of averted deaths based on infant mortality differentials by timing of diagnosis derived before the advent of universal screening [10]. Newly available estimates of the relative reduction in deaths associated with CCHD screening mandates based on a retrospective analysis of CCHD deaths in US states with universal screening policies offer evidence of a greater number of deaths averted [46] than had been projected by Peterson et al. [10] Using the direct estimates of averted deaths associated with mandatory CCHD screening policies from Abouk et al. [46] yields cost-effectiveness ratios more favorable for universal screening policies.

A comparative analysis of life expectancy among CCHD survivors relative to the general population can contribute evidence to improve future economic evaluations of CCHD screening. Where previous CEAs have assumed a normal life expectancy for individuals that through CCHD screening detection avoided CCHD-associated death, accounting for a shorter than average life expectancy will increase the net cost of screening by increasing the cost per life-year gained. Finally, previous CEAs have not accounted for CCHD-related expenditures associated with averted CCHD death in infancy. Accounting for such costs will also presumably increase the net cost of CCHD screening.

In conclusion, uncertainty remains regarding several parameters that are important to the analysis of the cost-effectiveness of CCHD screening. Nonetheless, the ability of universal CCHD screening to detect many newborns with CCHD who would otherwise have remained undiagnosed at the time of discharge—and at risk of severe morbidity and mortality—has been demonstrated [65–67].

## Supplementary Material

Supplement

## Figures and Tables

**Table 1:** Types of Critical Congenital Heart Disease and Associated International Classification of Disease, 10th Version (ICD-10) Diagnosis Codes.

CCHD Types	ICD-10 Codes
Aortic interruption or atresia or hypoplasia	Q25.4, Q25.2
Coarctation or hypoplasia of the aortic arch	Q25.1
D-transposition of the great arteries	Q20.3
Double-outlet right ventricle	Q20.1
Ebstein anomaly	Q22.5
Hypoplastic left heart syndrome	Q23.4
Pulmonary atresia	Q22.0
Single ventricle	Q20.4
Teratology of Fallot	Q21.3
Total anomalous pulmonary venous connection	Q26.2
Tricuspid stenosis and atresia	Q22.4
Truncus arteriosus	Q20.0

**Table 2:** Summary of data assumptions of micro-costing studies of critical congenital heart disease screening.

Study	Country and Jurisdiction	Screening Time (Minutes)	Screening Staff Type and Labor Cost per Infant	Type of Probes, and Equipment/Supply Cost per Infant	Total Screening Cost per Infant
Knowles et al. [13,14]	United Kingdom	2.0	Senior house officer £1.54 $2.20 (USD)	Reusable £1.28 $1.83 (USD)	£2.82 (2000–2001 prices) $4.03 (USD)
Roberts et al. [12]	United Kingdom	6.9	Midwives Not reported	Reusable Not reported	£6.24 (2009 prices) $8.80 (USD)
Peterson et al. [21]	United States New Jersey	9.1	Registered nurses $7.36	Mixed types $6.83	$14.19 (2011 prices)
Kochilas et al. [22]	United States Minnesota	5.5	Nursing staff $3.32	Reusable $1.82	$5.10 (2012 prices)
Reeder er al. [23]	United States Utah (two hospitals)	8.4	Medical assistants and nurses $2.60	Disposable $21.92	$24.52 (2014 prices)
		9.8	Nursing assistants $2.35	Reusable $0.25	$2.60 (2014 prices)

Note: all currency conversions were calculated using the purchasing power parity exchange rate for the year of the original cost estimate. Source: https://data.oecd.org/conversion/purchasing-power-parities-ppp.htm.
