# Supplementary material for: Cost and Cost-Effectiveness Assessments of Newborn Screening for Critical Congenital Heart Disease Using Pulse Oximetry: A Review
Source: Int J Neonatal Screen. Author manuscript; Available in PMC 2018 Jan 25. (PMC5784211; doi:10.3390/ijns3040034)
Supplement: Supplement [file NIHMS931597-supplement-Supplement.pdf]

Supplement: Projected Reductions in Infant Cardiac Deaths in the United States with Universal CCHD Screening Mandates

Abouk et al. [46] conducted an observational difference-in-difference analysis of Period Linked Birth-Infant Death Data files for 26,546,503 million US births from January 1, 2007 through June 30, 2013, aggregated by month and state of birth with deaths observed through December 31, 2013. State policies were classified as mandatory if a mandate to birthing centers was in place on the first day of the month. Non-mandatory policies included voluntary policies and mandates that were not yet implemented. As of June 1, 2013, eight states had implemented mandatory screening policies, five states had non-mandatory screening policies, and nine states had adopted but not yet implemented mandatory screening policies.

Abouk et al. calculated numbers of early infant deaths (between 24 hours and six months of age) coded for CCHD and for other/unspecified congenital cardiac causes for each state-month birth cohort for states with different CCHD screening policies. Most of the deaths in the other/unspecific CHD category were coded for unspecified defects; an unknown proportion may have had a critical defect. The authors estimated Poisson regression analyses of counts of early infant deaths per state-month birth cohort. Regression coefficients were then converted to proportional reductions.

The main results were that adjusted early infant deaths from CCHD through December 31, 2013 decreased by 33.4% (95% CI 10·6%–50·3%) after states implemented mandatory CCHD screening compared to prior periods and states without screening policies, in addition to a 21.4% (95% CI 6·9%–33·7%) decrease in other or unspecified cardiac deaths. No significant decrease was associated with non-mandatory screening policies.

Abouk et al. also projected the potential reduction in annual early infant deaths from CCHD and other CHDs if all states had universal CCHD screening policies relative to no CCHD screening. They multiplied 3.978 million births in the United States in 2015 [68] by the products of the baseline early infant death rates and the percentage declines associated with screening policies. They projected that if all states were to implement mandated universal screening, early infant deaths caused by recognized CCHD were projected to decline by between 38 and 181 deaths per year relative to no screening. The point estimate of 120 avoided deaths is the product of the proportionate decline in the CCHD early infant death rate (33.4%), the average of the CCHD death rate in the two years prior to the year of the first CCHD screening policy, the baseline CCHD early infant death rate for states that implemented mandates, and the annual number of births divided by 100,000. Similarly, the projected absolute reduction in the annual number of early infant cardiac deaths with other (non-CCHD) CHD codes is 117, with a range between 38 and 185.
